# The impact of smoking on immunological response to SARS-COV 2: a nationwide seroepidemiological study

**DOI:** 10.1093/eurpub/ckac131.143

**Published:** 2022-10-25

**Authors:** S Sahakyan, L Musheghyan, D Muradyan, Z Sargsyan, V Petrosyan, V Khachadourian, A Harutyunyan

**Affiliations:** Turpanjian College of Health Sciences, American University of Armenia, Yerevan, Armenia

## Abstract

**Background:**

Smoking influences cellular and humoral immune responses and affects the immune system by increasing inflammation and decreasing activity against infections. The current study investigates the association between smoking and immunological response to SARS-CoV-2 in the Armenian population.

**Methods:**

We performed a nationwide cross-sectional seroepidemiological study among the adult population (≥18 years old) in Armenia. We used a multi-stage cluster random sampling to recruit participants from the capital city and all regions of Armenia. We invited selected participants to primary healthcare facilities to provide blood samples for antibody testing followed by a phone survey on demographic characteristics, smoking status, and other variables. Logistic regression analysis was used to test the relationship between smoking and having SARS-CoV-2 antibodies adjusted for other covariates.

**Results:**

3483 people participated in the study (71% women). The total sample included 16.8% current smokers (n = 571), 8.6% past smokers (n = 294) and 76.4% never smokers (n = 2538). The prevalence of SARS CoV-2 antibodies among current smokers was statistically significantly lower as compared with never smokers (46.9% vs 73.4%, p-value<0.001). In the multivariable logistic regression model, the odds of having SARS CoV-2 antibodies among the current smokers was 70% lower (OR 0.30, 95%CI: 0.22; 0.40) compared to never smokers, when adjusted for demographic factors and the time of PCR diagnosis of COVID-19. No statistically significant difference was found between past smokers and having SARS CoV-2 antibodies.

**Conclusions:**

In addition to being a risk factor for various chronic diseases, smoking weakens immune response to infectious diseases, including COVID-19, worsening the outcomes. The significantly lower level of antibody prevalence among smokers with previous PCR confirmed COVID 19 implies a poorer immune response to the infection and not a lower risk of getting the infection.

**Key messages:**

• Smoking weakens immune response and contributes to a higher burden of infectious diseases, such as COVID-19.

• Lower level of antibody prevalence among smokers indicates a poorer immune response to the infection rather than a lower risk of getting the infection.

